# Brain Synchronization and Multivariate Autoregressive (MVAR) Modeling in Cognitive Neurodynamics

**DOI:** 10.3389/fnsys.2021.638269

**Published:** 2022-06-24

**Authors:** Steven L. Bressler, Ashvin Kumar, Isaac Singer

**Affiliations:** ^1^Center for Complex Systems and Brain Sciences, Boca Raton, FL, United States; ^2^Department of Psychology, Florida Atlantic University, Boca Raton, FL, United States

**Keywords:** MVAR modeling, synchronization, neurocognitive networks, Granger causality, cognitive neurodynamics

## Abstract

This paper is a review of cognitive neurodynamics research as it pertains to recent advances in Multivariate Autoregressive (MVAR) modeling. Long-range synchronization between the frontoparietal network (FPN) and forebrain subcortical systems occurs when multiple neuronal actions are coordinated across time (Chafee and Goldman-Rakic, 1998), resulting in large-scale measurable activity in the EEG. This paper reviews the power and advantages of the MVAR method to analyze long-range synchronization between brain regions (Kaminski et al., 2016; Kaminski and Blinowska, 2017). It explores the synchronization expressed in neurocognitive networks that is observable in the local field potential (LFP), an EEG-like signal, and in fMRI time series. In recent years, the surge in MVAR modeling in cognitive neurodynamics experiments has highlighted the effectiveness of the method, particularly in analyzing continuous neural signals such as EEG and fMRI (Pereda et al., 2005). MVAR modeling has been particularly useful in identifying causality, a multichannel time-series measure that can only be consistently computed with multivariate processes. Due to the multivariate nature of neuronal communication, multiple non-linear multivariate-analysis models are successful, presenting results with much greater accuracy and speed than non-linear univariate-analysis methods. Granger’s framework provides causal information about neuronal flow using neural time and frequency analysis, comprising the basis of the MVAR model. Recent advancements in MVAR modeling have included Directed Transfer Function (DTF) and Partial Directed Coherence (PDC), multivariate methods based on MVAR modeling that are capable of determining causal influences and directed propagation of EEG activity. The related Granger causality is an increasingly popular tool for measuring directed functional interactions from time series data.

## Introduction

Complex systems are of central interest to cognitive neuroscience. Any system composed of multiple moving parts is complex. The brain is a complex system. So is the cerebral (neo)cortex, that part of the brain most essential for cognition. Synchronization is a hallmark of complex systems. Brain synchronization within large-scale networks has been a primary research topic for many cognitive neuroscientists over the course of several decades. Large-scale coordination concerns brain synchronization within the broader context of coordinated structures in nature, a subject of study in physics, biophysics, and other disciplines. Directed functional connectivity is synchronization in regard to fMRI studies, and this paper discusses mathematical models for measuring directed functional connectivity. The mentioned directed functional connectivity is a key means of measuring synchronization within large-scale neocortical networks, or neurocognitive networks. Neurocognitive networks are defined as large-scale, distributed, interconnected systems of brain areas in the central nervous system that are joined together to perform a particular cognitive task (Bressler and Menon, 2010). Neurocognitive networks require a binding mechanism to function. The brain and cerebral cortex are complex systems that use binding mechanisms. They can be studied by applying directed functional connectivity methods because brain areas are linked by axon bundles, containing axons on which action potentials all travel in the same direction. This paper seeks to provide a review of Multivariate Autoregressive (MVAR) modeling in cognitive neurodynamics. Previous studies using MVAR modeling to analyze continuous neural signals are detailed, and further conclusions are drawn regarding given data and long-term implications.

Synchronization within neurocognitive networks concerns neural oscillations, brain activity that occurs in different brain areas that may or may not be spatially distant. Neural oscillations are unique in that they only display frequency, amplitude, and phase. In fact, when two or more neural oscillations are in synchronization, the exhibited frequency is always identical, a characteristic feature of the activity of neural oscillations. Amplitude and phase synchronization are both observed in the brain. Each is a possible mechanism of synchronization and may serve as a binding mechanism between neuronal populations within brain regions.

Considerable efforts have been made to model the directed functional connectivity in neurocognitive networks, and multiple mathematical models have been proposed to better interpret synchronization as a binding mechanism for neuronal populations in the brain. Of the models that have thus been developed, multivariate models are far more accurate and statistically significant than univariate non-linear models. Among other factors, the multi-dimensional nature of neuronal communication means that multivariate models are more ideal for interpreting directed functional connectivity and synchronization within the brain. Of the mathematical models that measure directed influences and causality within neurocognitive networks, Granger causality is the most basic and widely used, and is the most widely applicable to statistical contexts concerning causal influence within broader networks.

Granger’s framework has been expanded on. Both partial directed coherence (PDC) and directed transfer function (DTF) exclusively interpret directed influences between time series in the multivariate framework (Faes et al., 2013). They both differ from Granger causality. PDC, a method introduced by Baccalá and Sameshima (2001), is an expansion of Granger causality that normalizes terms in the frequency domain by the total outflow at a site. DTF, a method introduced by Kaminski and Blinowska, normalizes frequency-domain terms by the total inflow at a site. It is an alternative causality model that normalizes directed influences by the sum of transfer functions entering the site, as opposed to the sum of transfer functions leaving a site that PDC measures. In most neural contexts, MVAR models using non-instantaneous effects are utilized to interpret rhythms. MVAR models, with and without instantaneous effects, are elaborated upon in the following sections.

## Methods

### Visuomotor Experiments in Macaque Monkeys

Macaque monkeys performed a visuomotor task with a GO/NO-GO response (Brovelli et al., 2004). Microelectrodes measured surface-to-depth Local Field Potentials (LFPs) from 15 sites across one hemisphere in the monkey’s neocortex. During the task, the monkey depressed and held a lever in a random interval of 0.12 to 2.2 s. On GO trials, the monkey was rewarded with water if it released the lever within 500 msec after stimulus onset. Extracted data were preprocessed by selection and removal of trials containing contaminated or incorrect behavioral responses. Correct GO-trial LFP recordings were combined to result in approximately 900 trials per monkey. The data were then interpreted using MVAR spectral analysis (Geweke and Singleton, 1980; Geweke, 1982). Model coefficients were estimated by treating the analyzed LFP data as realizations of a common stochastic process.

**FIGURE 1:**
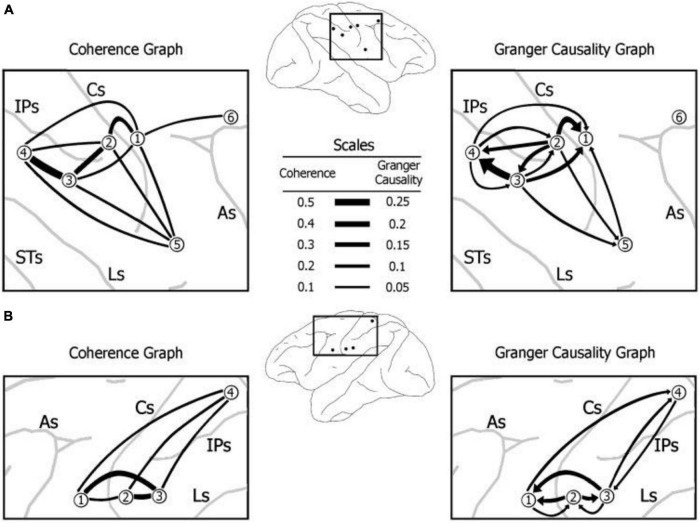
Prestimulus beta-frequency coherence and (conditional spectral) Granger causality maps derived from the sensorimotor cortices of two monkeys **(A,B)**. A self-generated hand press (–1250 to –500 msec) cued the monkey that a visual stimulus (0 msec onset; 100 msec duration) was soon to appear on a visual display screen. The stimulus was subsequently perceptually discriminated as part of a visual pattern discrimination task (water reward given on response trials 500 msec after stimulus onset) (Bressler et al., 1993). In each case, the pattern of synchronization (coherence) and Granger causality of beta-band oscillations from primary and secondary somatosensory and motor cortices consistent with execution of the hand press cue: somatosensory input is fed to the primary somatosensory cortex and motor output is transmitted from the primary motor cortex to the motor spinal cord to the hand muscles to execute the hand press. Sulci: Cs – Central; As – Anterior; Ls – Lunate; STs – Superior Temporal; IPs – IntraParietal (Figure modified from Brovelli et al., 2004).

Power, coherence, and relative phase spectral measures were estimated from the MVAR spectral matrix. The largest power peak by far was in the beta frequency range. Coherence and Granger causality spectra also contained prominent peaks in the beta frequency range. Coherence spectra were then analyzed for synchronized beta oscillations. A permutation distribution was created, and significance values were corrected using Dunn’s multiple comparison procedure. Granger causality spectral analysis was also used to identify the predictability and relative strengths of influence at various cortical sites of the monkeys. Peak values and frequencies were identified and listed for each coherence and Granger causality spectrum at *p* < 0.005 (Figure 1).

### FPN Synchronization During Working Memory in Macaque Monkey

**FIGURE 2:**
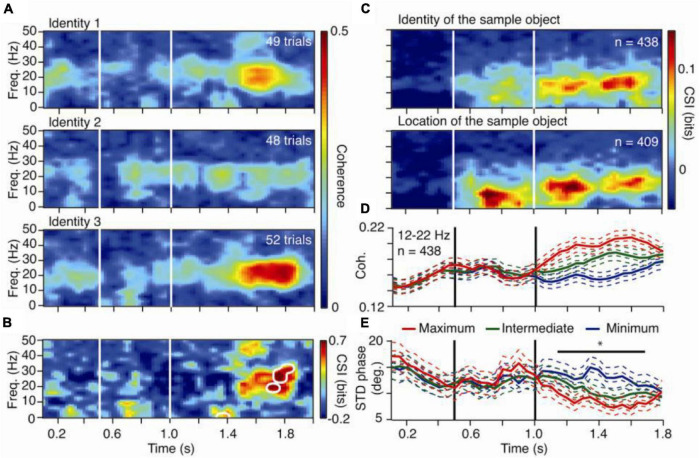
Content specific fronto-parietal synchronization during working memory in the macaque monkey. **(A)** Time-frequency coherence spectra for an LFP pair for three sample objects presented at one screen location. **(B)** Coherence selectivity index as a function of time and frequency [CSI(t,f)] for the same pair showing significant selectivity (significance threshold at *p* < 0.02 indicated by white contours) during the delay period. **(C)** Median value of [CSI(t,f)] for LFP pairs showing selectivity for the sample identity (upper) and location (lower) during the delay. **(D)** Mean rank-ordered coherence (+/– SEM) in the 12–22 Hz band for the same identity selective pairs as in the upper plot of **(C)**. **(E)** Mean standard deviation of the relative phase (+/– SEM) in the 12–22 Hz band for the same identity selective pairs as in the upper plot of **(C)**. In plots **(D,E)**, the two SEMs were calculated with the number of pairs or sessions as the degree of freedom.

Macaque monkeys performed a delayed match-to-sample task, while frontoparietal network (FPN) areas were monitored (Salazar et al., 2012). Two macaque monkeys matched the identity of a sample object, while recordings were made of broadband neuronal activity from 6 prefrontal cortical (PFC) and 6 posterior parietal cortical (PPC) sites (Figure 2). LFPs were recorded from monkeys A (over 27 days) and B (over 47 days). Time-frequency coherence and Granger causality spectra were computed for all FPN pairs (Figure 2). Pairs having significant spectra were identified, and peak values and frequencies were listed. Identity selectivity was also identified at each stimulus location.

Pairs with significant coherence selectivity index (CSI) were identified, and pairs having common CSI during the delay were grouped. Mean and variance of relative phase and power of beta-range distributions were displayed. To determine the relation between cortical regions and FPN synchronization in working memory activity, fronto-parietal pairs showing significant CSIs were sorted. To determine cortical regions showing dominant activity in working memory, Granger causality evaluated the prediction that FPN synchronization is governed by synaptic influences in the PFC, originating in the PPC (Chauvette et al., 2012) (Figure modified from Salazar et al., 2012).

### fMRI Blood-Oxygen-Level-Dependent Studies of Top-Down Influence in Human Neocortex

**FIGURE 3:**
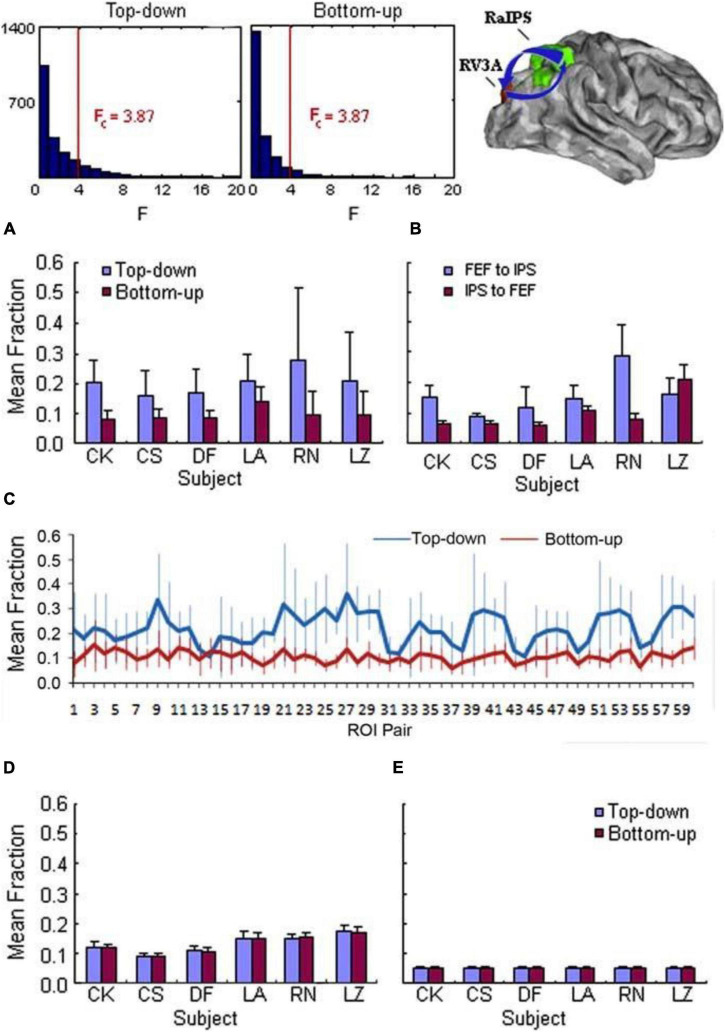
Top row. Top-down (top left) and bottom-up (top middle) Granger causality *F*-statistic histograms for a representative ROI pair, right aIPS and right V3A, in one subject. The critical value of *F* is 3.87 for significance (*p* < 0.05) in both directions. A larger fraction of the total number of voxel pairs (1064) had significant *F* statistics in the top-down (16.9%) than in the bottom-up (8.7%) direction. The schematic diagram (top right) showed Granger causality represented as arrows in the two directions between these ROIs on a standard brain image. Arrow thickness corresponds to the significant fraction, representing Granger causality strength. Bottom Top-down Granger causality before correct versus incorrect performance. **(A)** Grid specifying the significance of correct versus incorrect performance difference in a group analysis of top-down Granger causality: *p < 0.05, **p < 1.0 × 10^10^, ***p < 1.0 × 10^30^; white, not significant. Each cell represents Granger causality from the row-labeled ROI to the column-labeled ROI. **(B)** The fraction of significant top-down Granger causality from right aIPS to left VP was significantly greater before correct (blue) than incorrect (red) performance in five of six subjects [Figure modified from Bressler (2018)].

Human subjects performed a visual anticipation task designed to test the hypothesis that prefrontal and parietal cortices contain control areas that send top-down signals to visual cortex to modulate activity there and instantiate visual anticipatory attention (Bressler et al., 2011; Figure 3).

### fMRI Blood-Oxygen-Level-Dependent Studies in the Human Dorsal Anterior Cingulate Cortex

**FIGURE 4:**
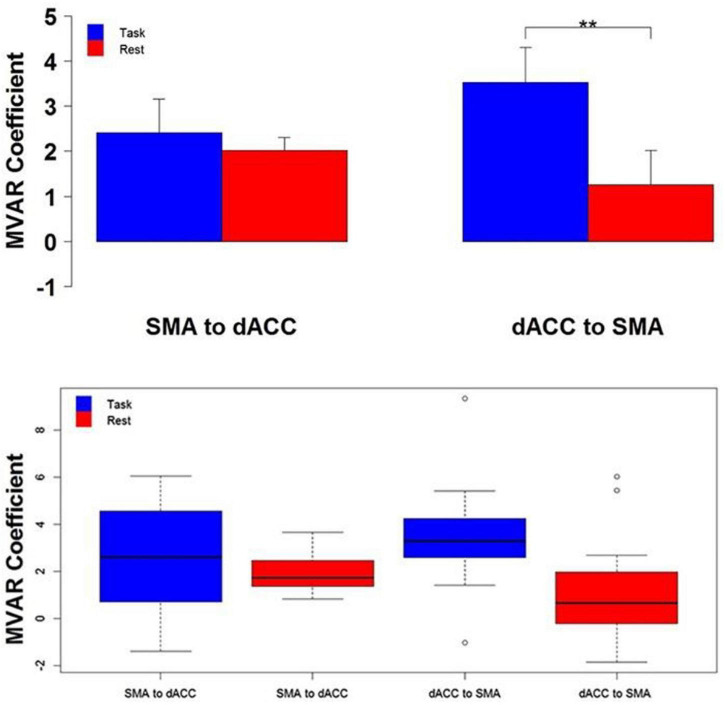
Top The group mean and standard error of influence in both directions between the 2 ROIs (SMA to dACC, dACC to SMA) for Task (blue) and Rest (red) conditions. *Post hoc* paired *t*-tests revealed that the influence from dACC to SMA was significantly greater for Task than Rest, but that the influence from SMA to dACC did not significantly differ (**p < 0.01). Bottom The distribution across subjects for both directions between the 2 ROIs (SMA to dACC, dACC to SMA) and for Task (blue) and Rest (red) conditions, as box plots. Note that the distribution for the influence from dACC to SMA during the Task is both more compact than and elevated above the distribution during Rest (Figure modified from Asemi et al., 2015).

Eleven adolescents participated in the study under proper ethical guidelines (Asemi et al., 2015; Figure 4). Participants were instructed to tap their right-hand forefinger as quickly as possible in response to a white flashing visual stimulus. fMRI Blood-Oxygen-Level-Dependent (BOLD) data were collected continuously across the study’s conditions. A complete BOLD scan lasted 6.83 s. Functional volumetric images were preprocessed with SPM5 under standard protocol (Friston et al., 1995). Regions of Interest (ROIs) were dorsal anterior cingulate cortex (dACC), Supplementary Motor Area (SMA) and primary motor cortex (M1) in the left hemisphere. Eigenvariate time series were extracted from voxels centered in these ROIs from an effects of interest contrast. Values were averaged and the average eigenvalue time series underwent analysis. ROI time series were preprocessed by *z*-value normalization and outlier rejection.

Pearson product-moment correlation coefficients were computed between the dACC fMRI BOLD time series and that of SMA and M1 for each participant. DFC was then estimated from MVAR models. Correlations were compared between task and rest by the parametric paired *t*-test and the non-parametric Mann-Whitney-Wilcoxon signed-rank test.

### Existing Multivariate Autoregressive Models

#### Granger Causality

The general unrestricted equation of Granger causality is as follows: $y\,\left(t\right)=\sum_{i=1}^{\infty }a_{i}\,y\,\left(t-i\right)+\sum_{j=1}^{\infty }\beta_{j}\,x\,\left(t-j\right)+c_{2}+v_{2}\,t$. Granger causality is a popular multivariate model that provides causal interactions from time-series data. Granger causality is the main mathematical method by which dependencies on multiple variables can be investigated as opposed to undirected influences (Seth et al., 2015). Causal influences are estimated using the conditional Granger causality index (CGCI). Granger causality is often used in conjunction with Dynamic Causal Modeling (DCM), a non-linear system that utilizes the Bayesian Model Comparison. Despite early controversies regarding the full potential to capture causal mechanisms within functional neural circuits, Granger causality has been largely characterized as an accurate and reliable method to measure directed influence among the neural circuits that underly behavior, cognition, and perception. A recent extension of Granger causality is proving popular (West et al., 2020).

#### Multivariate Autoregressive Model Without Instantaneous Effects

The Multivariate Autoregressive Model without Instantaneous Effects is described by the following equation: $\text{X}\,\left(\text{n}\right)=\sum_{k=1}^{p}A\,\left(k\right)\,X\,\left(n-k\right)+U\,\left(n\right)$, where p is the model order, A(k), *k* = 1,…, p, are M × M matrices contain the elements A_*ij*_(k) (Erla et al., 2009). MVAR models without instantaneous effects describe causality influences with lag and are generally less popular than MVAR models with instantaneous effects.

#### Multivariate Autoregressive Model With Instantaneous Effects

The Multivariate Autoregressive Model with Instantaneous Effects is described by the following equation: $\text{X}\,\left(\text{n}\right)=\sum_{k=0}^{p}B\,\left(k\right)\,X\,\left(n-k\right)+W\,\left(n\right)$. The input noise W(n) = [W1(n),…, Wm(n)] ^T is a vector of zero-mean uncorrelated processes with diagonal covariance matrix ∑*W* (Erla et al., 2009). MVAR models with instantaneous effects describe causality effects with the instantaneous terms included, as opposed to MVAR models without instantaneous effects. MVAR models with instantaneous effects are generally more popular than MVAR models without instantaneous effects.

#### Partial Directed Coherence Derived From Granger Causality

Partial Directed Coherence is an MVAR model proposed by Baccalá and Sameshima (2001) that comprises a frequency-domain method for Granger causality. The Partial Directed Coherence from channel j to i is represented by the following equation: $P_{i\,j}\,\left(f\right)=\frac{A_{i\,j}\,\left(f\right)}{\sqrt{a_{j\,\wedge^{*}\,\left(f\right)}\,a_{j}\,\left(f\right)}}$ (Baccalá and Sameshima, 2001; Sameshima and Baccalá, 2014). Partial Directed Coherence displays directed influences between channels, normalized by the outflows from the j-th site.

#### Directed Transfer Function Derived From Granger Causality

Directed Transfer Function (DTF) is an MVAR model proposed by Kaminski and Blinowska (Kaminski and Blinowska, 1991; Kus et al., 2004) that is closely related to the PDC model. The DTF from channel j to i is represented by the following equation: $D\,T\,F_{j\,i}\,\left(f\right)=\frac{{\left|H_{i\,j}\,\left(f\right)\right|}^{2}}{\sum_{m=1}^{k}{\left|H_{i\,m}\,\left(f\right)\right|}^{2}}$ (Kaminski and Blinowska, 1991). DTF is normalized by the sum of inflows to the i-th site. The model is applicable to a wide variety of neurophysiological contexts. To distinguish direct from indirect influences, direct Directed Transfer Function (dDTF) is utilized. Direct Directed Transfer Function from channel j to channel i is represented by the following equation: $d\,D\,T\,F_{j\,i}\,\left(f\right)=F_{i\,j}^{2}\,C_{i\,j}^{2}\,\left(f\right)$ (Korzeniewska et al., 2003). dDTF has been used, for example, to identify relations between primary motor sites.

### Inherent Mechanisms of Multivariate Autoregressive Models

The MVAR models discussed are subsets of directed frequency-domain influences, under the general set of functional connectivity metrics. Functional connectivity is regarded as the incidence of statistically related neurophysiological events occurring in spatially distant regions of the brain (Pourahmadi and Noorbaloochi, 2016). Ongoing research on functional connectivity seeks to uncover large-scale neural circuits responsible for executive function and behavior. Modeling of brain activity is an increasingly critical undertaking in order to fully understand the mechanisms of brain function and connectivity. Although there have been considerable efforts to investigate and understand brain rhythms, future research will be needed to fully understand the mechanisms of brain activity, and in particular, calculated mean-power-spectra-displayed beta oscillatory activity at peaks surrounding 20 Hz, the oscillations being apparent in large-scale cortical networks. While classical univariate analysis is useful in particular contexts, multivariate time series analysis more fully captures the dynamic nature of brain connectivity and communication. The nature of neuronal connectivity is such that multiple inputs over a specified frequency and time-domain must be considered, thus justifying the need for MVAR modeling.

The methods of the discussed research are mainly mathematical. The following Multivariate Autoregressive Model is used to analyze continuous neural signals in the form of EEG and fMRI: *X*_*i*,*t*_ = *a*_*i*, 1, 1_*X*_1,*t*−1_ + *a*_*i*,1,2_*X*_1,*t*−2_ + … + *a*_*i*,1,*m*_*X*_1,*t*−*m*_ + *a*_*i*,2,1_*X*_2,*t*−1_ + *a*_*i*,2,2_*X*_2,*t*−2_ + … + *a*_*i*,2,*m*_*X*_2,*t*−*m*_ + … + *a*_*i*,*p*,1_*X*_*p*,*t*−1_ + *a*_*i*,*p*,2_*X*_*p*,*t*−2_ + … + *a*_*i*,*p*,*m*_*X*_*p*,*t*−*m*_ + *e*_*i*,*t*_. This equation can be further stated in matrix form as the following: *X*_*t*_ = *A*_1_*X*_*t*−1_ + … + *A*_*m*_*X*_*t*−*m*_ + *E*_*t*_, where *X*_*t*_ = [*X*_1*t*_,*X*_2*t*_,…,*X*_*pt*_]∧*T* are *p* data channels, *m* is the model order, *A*_*k*_ are *p × p* coefficient matrices, and *E*_*t*_ is the white noise residual error process vector. Spectral Analysis is then conducted from MVAR modeling using the Spectral Matrix *S*(*f*) = < *X*(*f*)*X*(*f*)^*^ > = *H*(*f*)∑H*(*f*) where * denotes matrix transposition and complex conjugation; ∑ is the covariance matrix of *E*_*t*_; and $H\,\left(f\right)=\left(\sum_{k=1}^{m}A_{k}\,e^{-2\,\pi \,i\,k\,f}\right)\,\wedge -1$ is the transfer function of the system. The Power Spectrum of channel k is *S*_*kk*_(*f*) which is the *k^th^* diagonal element of the spectral matrix. The parameters of the mentioned MVAR model have been determined by solving the multivariate Yule-Walker equations:$\sum_{k=1}^{m}A\,{\left(k\right)}^{*}\,R_{x}\,\left(i-k\right)=-R_{x}\,\left(i\right),$ where ≤*i*≤*m*.

## Results

### Visuomotor Experiments in Macaque Monkeys

The calculated BOLD mean power spectra displayed beta oscillatory activity at peaks surrounding 20 Hz mean. Coherence and Granger causality spectra also displayed peaks near 20 Hz. Coherence spectra displayed synchronization of oscillations. Granger causal (GC) influences between cortical sites were mediated by beta range oscillations, as illustrated by the pronounced peaks surrounding 20 Hz in GC spectra. Coherence spectra were tested to identify significant beta peaks, a classic sign of synchronized beta LFP oscillations. Ultimately, coherence values were largest in three brain regions: the primary motor cortex, primary somatosensory cortex, and an area inferior to the intraparietal sulcus. GC oscillations within the beta oscillatory network were determined. GC spectra and coherence were closely linked, although GC influences were not found to be linked with the time delay values from phase spectra. Inferior posterior parietal sites exerted greater influence on the GC relations of primary motor sites.

### Frontoparietal Network Synchronization During Visual Working Memory in Macaque Monkeys

The calculated CSI and WGC values demonstrated that FPN synchronization during visual working memory tasks is widespread, content-specific, and task-dependent during the delay period. PPC influences dominate the synchronization patterns (Verhoef et al., 2011). The findings of this experiment are generally consistent with past studies concerning the spatial attention modulation of inter-areal coherence (Verhoef et al., 2011). Short-term memories are represented as patterns that are specific to stimuli and are characteristic of synchronized activity widely distributed throughout the FPN (Tallon-Baudry et al., 2001). Ultimately, it was concluded that the FPN exerts top-down control of and transmits behavioral influences to the visual cortex during the delay period of the given task (Bressler and Richter, 2015; Richter et al., 2018). The beta frequency band was observed to contain the main spectral peak in the delay period, and the beta GC from the frontal cortex was especially strong during the delay period.

### fMRI Blood-Oxygen-Level-Dependent Studies in the Human Neocortex

Top-down influences from the frontal eye field and the intraparietal cortex to the visual cortex were found in a hemisphere of the human brain prior to an expected visual stimulus in relation to anticipatory visual spatial attention, suggesting that frontal and parietal control signals modulate sensory cortex (Bressler et al., 2011). Other site pairs showed bottom-up, but not top-down, influences.

### fMRI Blood-Oxygen-Level-Dependent Studies in the Human Dorsal Anterior Cingulate Cortex

Response data were not driven by frequency or periodicity, as covariance measures with frequency and periodicity did not have statistical significance. A statistically significant relationship was observed during Pseudo-random epochs for the relationship between the percentage of missed responses and age, suggesting that the frequency of missed responses decreases with age. Measured correlations between dACC and SMA, and between dACC and M1, were greater in the Periodic condition than the Pseudo-random condition by both conducted paired *t*-tests and signed-ranked tests. This finding suggests that dACC activity is tightly related to both areas when predictable stimuli are present. Ultimately, results demonstrated greater DFC in the top-down direction than the bottom-up direction, indicating the presence of top-down motor control, a major component of many complex behaviors, exerted by the dACC in this task.

The employed mathematical models indicate with statistical significance that top-down synchronization exists in human brains. Furthermore, the results indicate that MVAR models are usable with statistical significance and accuracy to determine causal interactions among neuronal groups in neurocognitive networks. Brain synchronization has been found to be a binding mechanism within neurocognitive networks, as reaffirmed by past MVAR modeling studies in cognitive neuroscience research.

## Discussion

All four experiments tested the concept of brain synchronization as a binding mechanism within neurocognitive networks and provided evidence for top-down synchronization in monkey and human brains. Causal influences also suggested that top-down synchronization from the frontal cortex may compose the basis for predictive coding and selective attentional set in the brain. Isolation of sensory and motor areas in neurocognitive networks suggests that top-down FPN synchronization may contribute to human understanding and comprehension. The methods of MVAR modeling and GC are generally effective in measuring large-scale networks. Directional influences revealed by GC determine the electrical impulses of brain activity, especially when analyzing synchronous neural circuits and large-scale neuronal activity. These methods are particularly accurate when exogenous inputs direct intracerebral responses (Chang et al., 2012) but are widely applicable in all neurophysiological contexts. Modeling between cortical signals is necessary when determining electrical activity across cortical regions, thus explaining why univariate models are not as effective as multivariate methods are. MVAR modeling has provided evidence for synchronization and coordination in neurocognitive networks, as supported by numerous studies. Brain synchronization as a binding mechanism within neurocognitive networks continues to be heavily researched, and MVAR modeling has increased understanding of brain synchronization since the inception of these methods in neuroscience. MVAR models continue to evolve into an ever-expanding and more relevant method set of tools for analyzing neuronal communication, with new models being developed constantly. Despite recent advances in MVAR modeling (Pagnotta and Plomp, 2018), there remain numerous opportunities for growth in MVAR modeling of neuronal populations. First, Granger non-causality analysis depends on available model covariates, and thus is slightly limited in the method’s scope when analyzing causality large-scale networks (Pourahmadi and Noorbaloochi, 2016). Second, the basic GC only models linear functions, once again restricting the scientific scope of this method (Seth et al., 2015). Since most normal EEGs are locally linear, this is not a great problem. Although these are minor hinderances in the applicability of GC, on which most MVAR models are based, there rests an even greater potential for analyzing synchronization and neural communication should these hinderances be removed.

The concept of brain synchronization as a binding mechanism within neurocognitive networks continues to be studied in great detail. Research continues to uncover new aspects of synchronization and coordination within the human brain.

## Ethics Statement

The studies involving human participants were reviewed and approved by FAU HSC. The patients/participants provided their written informed consent to participate in this study. The animal study was reviewed and approved by FAU IACUC.

## Author Contributions

AK wrote the initial draft. SB conceptualized the review and edited the manuscript. All authors contributed to the article and approved the submitted version.

## Conflict of Interest

The authors declare that the research was conducted in the absence of any commercial or financial relationships that could be construed as a potential conflict of interest.

## Publisher’s Note

All claims expressed in this article are solely those of the authors and do not necessarily represent those of their affiliated organizations, or those of the publisher, the editors and the reviewers. Any product that may be evaluated in this article, or claim that may be made by its manufacturer, is not guaranteed or endorsed by the publisher.

## References

[B1] Asemi A. Ramaseshan K. Burgess A. Diwadkar V. A. Bressler S. L. (2015). Dorsal anterior cingulate cortex modulates supplementary motor area in coordinated unimanual motor behavior. *Front. Hum. Neurosci.* 9:309. 10.3389/fnhum.2015.00309 26089783 PMC4454840

[B2] Baccalá L. A. Sameshima K. (2001). Partial directed coherence: a new concept in neural structure determination. *Biol. Cybern.* 84 463–474. 10.1007/PL00007990 11417058

[B3] Bressler S. L. (2018). “Anticipatory top-down interactive neural dynamics,” in *Advances in Cognitive Neurodynamics*, eds Delgado-Garcia J. M. Sanchez-Campusano R. Pan X. Wang R. (Singapore: Springer).

[B4] Bressler S. L. Coppola R. Nakamura R. (1993). Episodic multiregional cortical coherence at multiple frequencies during visual task performance. *Nature* 366, 153–156. 10.1038/366153a0 8232553

[B5] Bressler S. L. Menon V. (2010). Large-scale brain networks in cognition: emerging methods and principles. *Trends Cogn. Sci.* 14 277–290. 10.1016/j.tics.2010.04.004 20493761

[B6] Bressler S. L. Richter C. G. (2015). Interareal oscillatory synchronization in top-down neocortical processing. *Curr. Opin. Neurobiol.* 31 62–66. 10.1016/j.conb.2014.08.010 25217807

[B7] Bressler S. L. Tang W. Sylvester C. M. Shulman G. L. Corbetta M. (2011). Top-down control of human visual cortex by frontal and parietal cortex in anticipatory visual spatial attention. *J. Neurosci.* 28 10056–10061. 10.1523/JNEUROSCI.1776-08.2008 18829963 PMC2583122

[B8] Brovelli A. Ding M. Ledberg A. Chen Y. Nakamura R. Bressler S. L. (2004). Beta oscillations in a large-scale sensorimotor cortical network: directional influences revealed by Granger causality. *Proc. Natl. Acad. Sci. U.S.A.* 101 9849–9854. 10.1073/pnas.0308538101 15210971 PMC470781

[B9] Chafee M. V. Goldman-Rakic P. S. (1998). Matching patterns of activity in primate prefrontal area 8a and parietal area 7ip neurons during a spatial working memory task. *J. Neurophysiol.* 79 2919–2940. 10.1152/jn.1998.79 9636098

[B10] Chang J. Y. Pigorini A. Massimini M. Tononi G. Nobili L. Van Veen B. D. (2012). Multivariate autoregressive models with exogenous inputs for intracerebral responses to direct electrical stimulation of the human brain. *Front. Hum. Neurosci.* 6:317. 10.3389/fnhum.2012.00317 23226122 PMC3510687

[B11] Chauvette S. Seigneur J. Timofeev I. (2012). Sleep oscillations in the thalamocortical system induce long-term neuronal plasticity. *Neuron* 75 1105–1113. 10.1016/j.neuron.2012.08.034 22998877 PMC3458311

[B12] Erla S. Faes L. Tranquillini E. Orrico D. Nollo G. (2009). Multivariate autoregressive model with instaneous effects to improve brain connectivity estimation. *Int. J. Bioelectromagnet.* 11 74–79.

[B13] Faes L. Erla S. Porta A. Nollo G. (2013). A framework for assessing frequency domain causality in physiological time series with instantaneous effects. *Philos. Trans. R. Soc. A* 371:20110618. 10.1098/rsta.2011.0618 23858484

[B14] Friston K. J. Holmes A. P. Poline J. B. Grasby P. J. Williams S. C. Frackowiak R. S. (1995). Analysis of fMRI time-series revisited. *Neuroimage* 2 45–53. 10.1006/nimg.1995.1007 9343589

[B15] Geweke J. F. (1982). Measurement of linear dependence and feedback between multiple time series. *J. Am. Stat. Assoc.* 77 304–313. 10.1080/01621459.1982.10477803

[B16] Geweke J. F. Singleton K. J. (1980). Interpreting the likelihood ratio statistic in factor models when sample size is small. *J. Am. Stat. Soc.* 75 133–137. 10.1080/01621459.1980.10477803

[B17] Kaminski M. Blinowska K. J. (1991). A new method of the description of the information flow in the brain structures. *Biol. Cybern.* 65 203–210. 10.1007/BF00198091 1912013

[B18] Kaminski M. Blinowska K. J. (2017). The influence of volume conduction on DTF estimate and the problem of its mitigation. *Front. Comput. Neurosci.* 11:36. 10.3389/fncon.2017.00036 PMC5427064 28553220

[B19] Kaminski M. Brzezicka A. Kaminski J. Blinowska K. J. (2016). Measures of coupling between neural populations based on Granger causality principle. *Front. Comput. Neurosci.* 10:114. 10.3389/fncom.2016.00114 27833546 PMC5080292

[B20] Korzeniewska A. Manczak M. Kaminski M. Blinowska K. J. Kasicki S. (2003). Determination of information flow direction among brain structures by a modified directed transfer function (dDTF) method. *J. Neurosci. Methods* 125 195–207. 10.1016/S0165-0270(03)00052-9 12763246

[B22] Kus R. Kaminski M. Blinowska K. (2004). Determination of EEG activity propagation: pair-wise versus multichannel estimate. *IEEE Trans. Biomed. Eng.* 51 1501–1510. 10.1109/TBME.2004.827929 15376498

[B23] Pagnotta M. F. Plomp G. (2018). Time-varying MVAR algorithms for directed connectivity analysis: critical comparison in simulations and benchmark EEG data. *PLoS One* 13:e0198846. 10.1371/journal.pone.0198846 29889883 PMC5995381

[B24] Pereda E. Quiroga R. Q. Bhattacharya J. (2005). Nonlinear multivariate analysis of neurophysiological signals. *Prog. Neurobiol.* 77 1–37. 10.1016/j.pneurobio.2005.10.003 16289760

[B25] Pourahmadi M. Noorbaloochi S. (2016). Multivariate time series analysis of neuroscience data: some challenges and opportunities. *Curr. Opin. Neurobiol.* 37 12–15. 10.1016/j.conb.2015.12.006 26752736

[B26] Richter C. G. Coppola R. Bressler S. L. (2018). Top-down beta oscillatory signaling conveys behavioral context in early visual cortex. *Sci. Rep.* 8:6991. 10.1038/s41598-018-25267-1 29725028 PMC5934398

[B27] Salazar R. F. Dotson N. M. Bressler S. L. Gray C. M. (2012). Content-specific fronto-parietal synchronization during visual working memory. *Science* 338 1097–1100. 10.1126/science.1224000 23118014 PMC4038369

[B28] Sameshima K. Baccalá L. A. (eds) (2014). *Methods in Brain Connectivity Inference Through Multivariate Time Series Analysis.* Boca Raton, FL: CRC Press.

[B29] Seth A. K. Barrett A. B. Barnett L. (2015). Granger causality analysis in neuroscience and neuroimaging. *J. Neurosci.* 35 3293–3297. 10.1523/JNEUROSCI.4399-14.2015 25716830 PMC4339347

[B30] Tallon-Baudry C. Bertrand O. Fischer C. (2001). Oscillatory synchronization between human extrastriate areas during visual short-term memory maintenance. *J. Neurosci.* 21:RC17. 10.1523/JNEUROSCI.21-20-j0008.2001 11588207 PMC6763859

[B31] Verhoef B. E. Vogels R. Janssen P. (2011). Synchronization between the end stages of the dorsal and the ventral visual stream. *J. Neurophysiol.* 105 2030–2042. 10.1152/jn.00924 21325682 PMC3094193

[B32] West T. O. Halliday D. M. Bressler S. L. Farmer S. F. Litvak V. (2020). Measuring directed functional connectivity using non-parametric directionality analysis: validation and comparison with non-parametric Granger causality. *Neuroimage* 218:116796. 10.1016/j.neuroimage.2020.116796 32325209 PMC7116477

